# Effects of bariatric surgery and dietary intervention on insulin resistance and appetite hormones over a 3 year period

**DOI:** 10.1038/s41598-023-33317-6

**Published:** 2023-04-13

**Authors:** Malgorzata M. Brzozowska, Michelle Isaacs, Dana Bliuc, Paul A. Baldock, John A. Eisman, Chris P. White, Jerry R. Greenfield, Jacqueline R. Center

**Affiliations:** 1 Endocrinology, The Sutherland Hospital, Caringbah, Australia; 2 Faculty of Medicine, UNSW Sydney, Sydney, Australia; 3 Garvan Institute of Medical Research, Healthy Ageing Theme, Darlinghurst, Australia; 4 Endocrinology, St Vincent’s Hospital Sydney, Darlinghurst, Australia; 5 School of Medicine, The University of Notre Dame Australia, Darlinghurst, Australia; 6 Prince of Wales Hospital, NSW Health Pathology, Randwick, Australia; 7 Endocrinology, Prince of Wales Hospital, Randwick, Australia

**Keywords:** Health care, Endocrinology, Endocrine system and metabolic diseases

## Abstract

To examine an impact of three types of bariatric surgery compared with dietary intervention (DIET), on concurrent changes in Homeostatic Model Assessment for Insulin Resistance (HOMA-IR) and appetite hormones over 3 years. Fifty-five adults were studied during phase of weight loss (0–12 months) and during weight stability (12–36 months) post intervention. Measurements of HOMA-IR, fasting and postprandial PYY and GLP1, adiponectin, CRP, RBP4, FGF21 hormones and dual-Xray absorptiometry were performed throughout the study. All surgical groups achieved significant reductions in HOMA-IR with greatest difference between Roux-en-Y gastric bypass and DIET (− 3.7; 95% CI − 5.4, − 2.1; p = 0.001) at 12–36 months. Initial (0–12 months) HOMA-IR values were no different to DIET after adjustment for the lost weight. During 12–36 months, after controlling for treatment procedure and weight, for every twofold increase in postprandial PYY and adiponectin, HOMA-IR decreased by 0.91 (95% CI − 1.71, − 0.11; p = 0.030) and by 0.59 (95% CI − 1.10, − 0.10; p = 0.023) respectively. Initial, non-sustained changes in RBP4 and FGF21 were not associated with HOMA-IR values. While initial rapid weight loss reduces insulin resistance, the enhanced secretions of PYY and adiponectin may contribute to weight-independent improvements in HOMA-IR during weight stability.

**Clinical trial registration**: Australian New Zealand Clinical Trials Registry (ANZCTR): ACTRN12613000188730.

## Introduction

Bariatric surgery achieves more substantial and sustained weight loss than conventional weight loss therapy with significant reductions in comorbid conditions and prolonged life expectancy^1^. Recent reports have confirmed long-term efficacy of Roux-en-Y gastric bypass (RYGB) and gastric sleeve (GS) surgeries, with maintenance of weight loss beyond 10 years^2,3^ through mechanisms of energy restriction associated with altered hormonal, neural and nutrient signaling^4^. Furthermore, data from observational and randomized controlled trials indicate that some bariatric procedures achieve partial or complete type 2 diabetes remission^5,6^. These metabolic benefits of bariatric surgery have been linked with the degree of weight loss and with hormonal changes after bariatric surgery^7,8^.

Murine data suggest that glucose homeostasis may be influenced by a gut–brain–liver axis in which gut-derived signals, acting centrally, regulate hepatic glucose production^8^. The physiological roles of gastrointestinal hormones in surgically induced diabetes remission have not yet been fully defined. Results from murine studies suggest that glucagon-like peptide 1 (GLP-1) may not be uniquely responsible for improved glucose homeostasis after bariatric procedures^9–11^. In a rat model of type 2 diabetes polypeptide tyrosine-tyrosine (PYY) hormone was associated with the reversal of impaired pancreatic islet function following RYGB surgery^12^.

Human studies have highlighted a relationship between changes in release patterns for gastrointestinal hormones GLP-1 and PYY and post-surgical changes in food intake, gluconeogenesis and weight loss during the first 12 months post RYGB surgery^13^. Exaggerated GLP-1 responses were linked to improved β-cell function during first 3 months after RYGB^14^. Interestingly, recent study demonstrated an important role of PYY hormone in long term restoration of impaired glucose-mediated insulin and glucagon secretion in bariatric subjects^15^.

Beneficial metabolic effects of bariatric surgery may also result from changes in circulating adipokines and hepatokines including adiponectin, retinol-binding protein 4 (RBP4), fibroblast growth factor-21 (FGF21) and C-reactive protein (CRP), which have anti-inflammatory, insulin-sensitizing and lipid lowering effects^16,17^. The underlying mechanisms for the altered secretion of these hormones are likely multifactorial in nature and mediated by significant weight reduction together with weight-loss independent mechanisms^16,18^.

Limited studies have examined the metabolic effects of altered gut hormones, adipokines and hepatokines beyond the initial phase of rapid weight loss and during the stages of postsurgical weight regain and stabilization. In the present manuscript we have explored the impact of three types of bariatric surgery: RYGB, GS and laparoscopic adjustable gastric banding surgery (LAGB) compared with DIET alone on changes in insulin resistance and glycaemic control. We have also examined the potential association between changes in insulin resistance and fasting and postprandial PYY and GLP1 hormones, adiponectin, RBP4, FGF21 and CRP over 3 years of follow-up.

The primary aim of this study was to examine the effects of three types of bariatric surgery and dietary intervention on changes in Homeostatic Model Assessment for Insulin Resistance (HOMA-IR) during initial phase of weight loss (0 to 12 months) and during weight stability (12 to 36 months) post interventions. Secondly, we investigated whether changes in gut hormones, adipokines and hepatokines during weight stability phase were associated with changes in insulin resistance.

## Materials and methods

The study was listed in Australian New Zealand Clinical Trials Registry (ANZCTR), 12613000188730. The protocol for this prospective, longitudinal, observational study of adult subjects with obesity, aged between 18 and 70 years, who underwent medically supervised dieting or bariatric surgery has been previously partly published^19^. However, the salient points are described below.

Inclusion criteria were body mass index (BMI) ≥ 30 kg/m^2^ and presence of obesity for at least 5 years, despite attempts to lose weight through other measures. Subjects were excluded if they were pregnant or planning a pregnancy within 2 years, were within 5 years post onset of menopause or had an active psychiatric problem that would limit adherence to the study protocol. Subjects were recruited from St Vincent’s, Royal North Shore and St George Hospitals in Sydney, Australia^19^.

The study participants were allocated to either their bariatric or dietary interventions based on their probability of diabetes remission criteria, which were congruent with DiaRem score. DiaRem grading system is based on four preoperative clinical variables being identified in the final scoring model: insulin use, participant age, HbA_1c_, and type of antidiabetic drugs used^20^.

The study was designed to enroll 60 study participants aiming for 15 study subjects in each study group. Sixty-two participants were recruited, of whom 7 withdrew after the baseline visit. The remaining 55 subjects were included in the analysis with RYGB (N = 7), GS (N = 21), LAGB (N = 11) and DIET-treated subjects (N = 16). The effects of these weight loss interventions on glycemic markers (fasting insulin, HOMA-IR) were only measured for 39 subjects who were not treated with diabetes agents (metformin for all treated patients).

**Figure 1:**
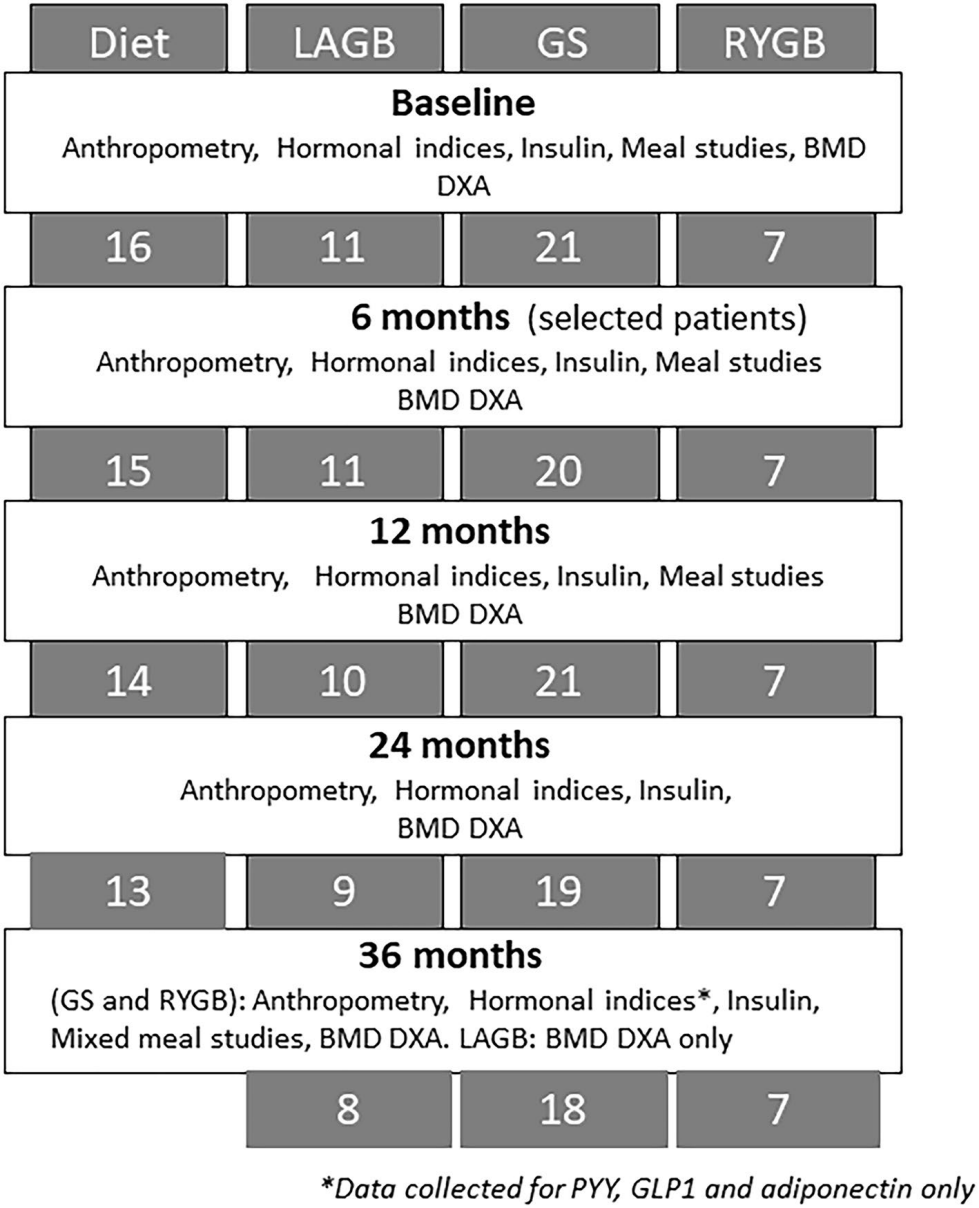
Flow of study participants and study procedures.

Assessments occurred at 7 time-points: at baseline and at 1-, 3-, 6-, 12-, 24- and 36-months post weight-loss interventions, see Fig. 1. Given lack of long-term weight loss and hormonal changes in the DIET groups, only surgical groups were assessed at 36 months with hormonal data being available for GS and RYGB groups.

The weight and body composition changes and changes in fasting and postprandial gut hormones PYY and GLP1 and adiponectin have been previously partly reported^19^. Body composition was measured with dual-energy X-ray absorptiometry (DXA; GE-LUNAR Prodigy instrument & analysis program).

Blood samples, drawn following an overnight fast, were analyzed for glucose, HbA1c, PYY, GLP1 and adiponectin at all time-points up to 36 months while fasting RBP4, FGF21 and CRP were measured up to 24 months post interventions. Fasting insulin and postprandial PYY and GLP1 were measured at baseline, 6-, 12-, 24- and 36-months. Insulin resistance was estimated using the homeostasis model assessment index-insulin resistance (HOMA-IR)^21^.

Ethical approval was obtained from the St Vincent’s Hospital Health Research Ethics Committee, Sydney HREC/09/SVH/64. Informed written consent was obtained from all participants. All methods were performed in accordance with the Declaration of Helsinki and in compliance with the National Health and Medical Research Council in Australia (NHMRC) National Statement on Ethical Conduct in Human Research (NHMRC, 2007).

### Biochemical variables

Blood glucose level was determined by the hexokinase method (Roche modular P analyzer, Roche Diagnostics, Castle Hill, NSW, Australia) and Hba1c by HPLC using the BioRad Variant II NU method (BioRad Laboratories, Gladesville, NSW, Australia). Serum insulin was measured by a chemiluminescent immunoassay (Advia Centaur analyzer, Siemens Diagnostics, North Ryde, NSW, Australia) until March 2012, when this was changed to an electrochemiluminescent immunoassay (Roche Modular E analyzer, Roche Diagnostics, Castle Hill, NSW, Australia). A correction equation was applied to results from the Siemens Centaur assay to give results comparable to those from the Roche assay^22^.

After an overnight 12 h fast, patients consumed a mixed meal (Ensure Plus 1757 kJ, 57% carbohydrate, 28% fat, 15% protein). As PYY increases 15 min after the meal and stays elevated for up to 6 h, the 90 min post mixed meal time point was chosen to estimate its postprandial increment^23^. For total PYY and GLP1 measurements, 0.260 mL Aprotinin (Sigma, Sydney, Australia) and 0.040 mL DPP-IV inhibitor (LINCO Research, Inc., St. Charles, USA) were added to EDTA blood collection tubes to prevent proteolysis. Serum and plasma samples were separated immediately by centrifugation at 4 °C and stored at – 80 °C until assayed.

Total PYY, GLP-1 (active) and adiponectin were measured using ELISA (EZHPYYT66K, EGLP-35 K and EZHADP-61 K; Millipore; St. Charles, MO, USA). The sensitivity of PYY assay was 6.5 pg/mL with inter- and intra-assay coefficients of variation (CVs) of < 3.7% while GLP-1 assay’s sensitivity was 2 pM with CVs of < 8%. The sensitivity of the adiponectin assay was 1.5 ng/mL with inter- and intra-assay CVs of < 2.4%. The antibody pair used in this assay is specific to Human Adiponectin with ability to selectively measure the analytes in the presence of other like components in the sample matrix up to 50 nM concentration. Serum hs-CRP, RBP4 and FGF21 were determined by immunoassays established by Antibody and Immunoassay Services, the University of Hong Kong. The observed intra and inter-assay CVs were 4.3% and 5.9% (hs-CRP), 2.4–3.6% and 2.6–4.4% (RBP4), and 4.0–5.0% and 3.5–10.2% (FGF21).

## Statistical analysis

Baseline characteristics of study subjects were analyzed according to intervention groups. Normally distributed variables were presented as mean (± SD) and non-normally distributed variables were presented as median (± interquartile range). Analysis of variance (ANOVA) and post-hoc pairwise Tukey honest significance difference test, or Kruskal–Wallis’ test and post-hoc pairwise Dunn’s test examined the comparisons between DIET and surgical groups for normally or non-normally distributed baseline data, respectively.

The non-normally distributed data such as fasting and postprandial PYY and GLP1 hormones, adiponectin, RBP4, FGF21, CRP underwent logarithmic transformation.

The differences in percentage change in examined hormones from their baseline to subsequent visits were analysed within study groups and compared to DIET by the linear mixed-effects regression model.

There were two distinct weight trajectories after bariatric procedures with rapid weight loss between 0 and 12 months followed by weight stability between 12 and 36 months^19^. Due to nonlinear changes in fasting insulin and HOMA-IR, which followed the patterns of weight loss, and to examine the changes in glucogenesis during the phase of weight stability, two separate random intercept mixed-effects models examined the short-term (i.e., until 12 months postoperative) and long-term (i.e., between 12 and 36 months postoperative) changes in fasting insulin and HOMA IR.

### Effects of bariatric surgery and DIET on changes in insulin resistance during 0–12 months and during 12–36 months.

Two random intercept linear mixed effects models analyzed changes in absolute values in outcome variables of fasting insulin and in HOMA-IR at 0-, 6-, 12-, 24- and 36-months (GS and RYGB) with co-variate variables being weight loss procedures and weight. Additionally, we examined whether HOMA- IR changes were related to surgical procedures independently of weight loss. Effects of bariatric surgeries on these continuous outcomes were expressed as difference in mean changes over time between specific bariatric surgery and DIET (95% confidence interval: corresponding *p* value).

### The association between changes in gut hormones, adipokines and hepatokines and changes in insulin resistance during 0–12 months and during 12–36 months

Changes in HOMA-IR were examined in separate models relative to changes in fasting and postprandial PYY and GLP1 and adiponectin during the two-time intervals beyond the effects of type of surgery and weight changes. An additional analysis assessed if hormonal changes in RBP4, FGF21 and CRP contributed to changes in HOMA-IR during the two weight loss intervals at 0–12 months and during 12–24 months. An exploratory analysis examined whether postoperative changes in gut hormones were associated with weight changes beyond the effects of treatment procedures.

In the primary analysis between 24 and 36 months, data for DIET group were imputed according to the trend derived from mixed effects models. Additionally, a sensitivity analysis was performed with imputed 3-year data for DIET subjects. These data were imputed using their own 2-year data and the changes between 1 and 2 years follow up, under an assumption of similar changes between the 2nd and 3rd years to those between the 1st and 2nd year post interventions.

All statistical analyses were performed using R software version 4.0.4 (2021-02-15) with P value < 0.05 considered significant.

## Results

### Baseline characteristics of study participants (see Table 1)

**Table 1:** Baseline characteristics.

Characteristics	DIET (n = 16)	Gastric band (n = 11)	Gastric sleeve (n = 21)	Gastric bypass (n = 7)	P values
Age (Years)	53.4 (8.6)	42.2 (13.0)	50.0 (11.7)	50.9 (7.2)	0.072
Gender: N (%)					0.11
Male	3 (18.8)	1 (9.1)	10 (47.6)	2 (28.6)	
Female	13 (81.3)	10 (90.9)	11 (52.4)	5 (71.4)	
Diabetes: N (%)	6 (37.5)	1 (9.1)	11 (52.4)	3 (42.9)	0.079
Fasting glucose (mmol/L)^^^	5.8 (5.1–6.9)	5.0 (4.5–5.2) *	5.1 (4.9–5.9)	5.3 (5.1–8.9)	**0.019**
HbA1c (mmol/mol)^^^	41 (37–53)	36 (34–40)	42 (38–46)	42 (36–58)	0.089
HbA1c (%)	5.9 (5.5–7.0)	5.4 (5.3–5.8)	6.0 (5.6–6.4)	6.0 (5.4–7.5)	
Insulin (mU/L)^^,#^	12.3 (9.8–14.4)	11 (6.9–24.4)	18.7 (15.7–26.8)	11.5 (5.0–24.8)	0.58
HOMA-IR^^,#^	2.7 (2.2–5.5)	2.4 (1.5–5.6)	4.6 (3.4–6.8)	6.0 (5.4–7.5)	0.84
Weight (kg)	110.9 (26.0)	105.0 (16.5)	125.3 (16.9) *	113.1 (15.9)	**0.036**
Lean mass (kg)	54.3 (9.3)	50.5 (7.1)	60.5 (7.9) *	54.2 (9.7)	**0.020**
Fat mass (kg)	49.1 (9.3)	51.3 (9.2)	56.8 (8.2)	55.3 (14.2)	0.12
Waist (cm)	122.1 (14.2)	110.9 (7.3)*	132.3 (12.6)*	119.7 (12.3)	**0.0002**
BMI (kg/m^2^)	38.1 (6.6)	37.7 (4.5)	42.5 (5.3)*	42.3 (7.7)	0.055
Fasting Adiponectin (ng/mL)^^^	6615 (4339–7609)	6407 (5152–9749)	6229 (4127–9339)	6029 (4318–11,187)	0.97
FGF21 (pg/mL)^^^	115.0 (62.9–164.0)	73.6 (51.7–91.7)	122.2 (31.4–210.5)	75.3 (66.0–180.0)	0.66
RBP4 (µg/mL)^^^	12.6 (10.4–15.1)	8.5 (7.3–9.7)*	9.5 (7.5–19.1)	18.6 (14.3–33.3)*	**0.0029**
CRP (µg/mL)^^^	5.1 (1.9–9.7)	5.5 (2.4–12.5)	11.9 (6.5–20.6)*	13.0 (3.8–17.6)*	**0.022**
PYY-fasting (pg/mL)^^^	104.0 (71.9–126.4)	64.8 (47.0–111.3)	104.9 (81.8–151.6)	83.7 (67.4–118.6)	0.27
Post-prandial (pg/mL)^^^	130.3 (108–166)	93.5 (63.8–158.8)	134.3 (105.2–178.3)	120.5 (77.4–133.2)	0.16
GLP1—fasting (pM/mL)^^^	8.1 (7.6–10.4)	6.4 (6.1–10.0)	7.5 (6.5–8.0)*	5.7 (5.0–6.1)*	**0.02**
Post-prandial (pM/mL)^^^	8.1 (7.8–12.1)	7.1 (6.1–9.3)	8.1 (7.5–8.7)	6.5 (5.3–7.0)*	**0.02**

Baseline characteristics of participants. Data are reported as count (%) for categorical data, mean (SD) for normally distributed continuous data and ^^^median (IQR) for non-normally distributed continuous data. ^#^Includes only those participants not taking diabetes medications (diet n = 9, LAGB n = 11, GS n = 12, RYGB n = 4). *Statistically significantly different from Diet group (post-hoc test). Significant values are in bold.

Baseline characteristics of the participants in each intervention group are shown in Table 1. GS subjects were heavier than DIET and DIET subjects had higher fasting glucose than LAGB group. There were also significant differences between DIET and surgical groups with regards to values of fasting and postprandial GLP1, CRP and RBP4 hormones.

### Changes in HbA1c over time following weight loss interventions

All three diabetic RYGB subjects, including a subject with an initial HbA1c of 101 mmol/mol (11.4%) reached HbA1c below 48 mmol/mol (6.5%) at 24 months and maintained HbA1c below 53 mmol/mol (7.0%) off diabetic medications, over 36 months after their surgeries. A major improvement in glycaemic control was noted in GS group, as 10 out of 11 (90%) diabetic participants, with 5 of them on metformin, maintained excellent diabetes control at 36 months, with their mean HbA1c of 40 mmol/mol (5.8%, SD = 1.3), which declined from their initial HbA1c of 51 mmol/mol (6.8%, SD = 1.2). At 24 months, DIET intervention led to HbA1c below 48 mmol/mol (6.5%) in half of 6 type 2 diabetes subjects on medical treatment.

### Changes in HOMA-IR and fasting insulin values during rapid weight loss (0–12 months) and during weight stability (12–36 months)

#### Changes in HOMA-IR and fasting insulin values within study groups

In the subgroup of subjects with and without diabetes, not taking diabetic medications, by 6 months (weight nadir), the HOMA-IR values and fasting insulin significantly declined for RYGB (*p* = 0.02), GS (*p* < 0.0001) and for DIET (*p* = 0.01). The improvement in HOMA-IR was sustained in RYGB and GS groups at 12 months, but not in the DIET group (*p* = 0.32). HOMA-IR did not change significantly from their baseline values in the LAGB group (*p* = 0.41).

During 12–36 months, during weight stability, the improvements in HOMA-IR and fasting insulin from their baseline values were maintained in RYGB (*p* < 0.0001) and in GS group (*p* < 0.0001) without such change in HOMA-IR and insulin in the LAGB group (*p* = 0.45).

At 24 months, the average HOMA-IR (RR 0.5–1.4) remained raised at 2.5 (SD = 0.52) for the LAGB group and at 4.6 (SD = 1.05) for the DIET group (Table 2). At 36 months, the mean HOMA-IR had normalized for the RYGB group at 0.8 (SD = 0.25) and for the GS group at 1.25 (SD = 1.15) (Supplementary Table 1).

**Table 2:** Random intercept linear mixed effect models examining comparisons between groups in fasting insulin and HOMA-IR over time.

	Annual change in insulin level (mU/L)			Annual change in HOMA-IR in level (units)		
	Difference in insulin level between surgical and DIET groups (95% CI)			Difference in HOMA-IR level between surgical and DIET groups (95% CI)		
	0–12 months	12–36 months		0–12 months	12–36 months	
Study groups			Sensitivity analysis			Sensitivity analysis
Diet	Ref.	Ref.	Ref.	Ref.	Ref.	Ref.
LAGB	− 3.7 (− 9.5, 2.1)	− **7.4 (**− **12.2, **− **2.5)**		− **2.6 (**− **4.9, **− **0.3)**	− **2.6 (**− **4.1, **− **1.1)**	
GS	− 1.8 (− 6.8, 3.1)	− **7.2 (**− **11.3, **− **3.1)**	− 2.6 (− 6.2, 1.0)	− **2.0 (**− **3.9, **− **0.03)**	− **2.6 (**− **3.9, **− **1.3)**	− **1.3 (**− **2.4, **− **0.2)**
RYGB	− **6.9 (**− **13.4, **− **0.5)**	− **12.4 (**− **17.7, **− **7.1)**	− **7.9 (**− **12.5, **− **3.3)**	− **2.8 (**− **5.3, **− **0.3)**	− **3.7 (**− **5.4, **− **2.1)**	− **2.4 (**− **3.8, **− **1.0)**

Data presented as annual change in Insulin level (mU/L/year) and HOMA-IR levels (units) with the corresponding 95% CI for two periods: baseline and 12 months, and between 12- and 36- months post intervention. RYGB Roux-en-Y gastric bypass, GS gastric sleeve, LAGB laparoscopic adjustable gastric banding. Boldface indicates statistical significance.

#### Comparisons between study groups in HOMA-IR values and in fasting insulin

During first 12 months, compared with DIET, all bariatric procedures led to significant reduction in HOMA-IR with the highest difference in RYGB group by − 2.8 (95% CI − 5.3, − 0.3; *p* = 0.04) (Table 2) while RYGB procedure led to significant reduction in fasting insulin level by − 6.9 mU/L (95% CI − 13.4, − 0.5; *p* = 0.04). The difference in HOMA-IR between DIET and surgical groups was no longer present after adjustment for weight loss.

During 12–36 months, in comparison with DIET, all bariatric procedures resulted in further significant reductions in insulin and HOMA-IR values with greatest differences in RYGB group by − 12.4 mU/L (95% CI − 17.7, − 7.1; *p* = 0.001) for the insulin level and by − 3.7 (95% CI − 5.4, − 2.1; *p* = 0.001) for HOMA-IR (Table 2). The HOMA- IR values decreased by 0.07 (95% CI 0.01, 0.14; *p* = 0.02) for each 1 cm decrease in waist diameter.

The sensitivity analysis, using 3-year imputed data for the DIET group, was consistent with both GS and RYGB procedures producing significant reductions in HOMA-IR levels between 12 and 36 months (Table 2).

#### The contribution of PYY, GLP1 and adiponectin to changes in insulin resistance and weight loss over time

The values of PYY, GLP1 and adiponectin over time are shown in Supplementary Table 1. During 0–12 months, when adjusted for the weight change and procedure, for every twofold increase in adiponectin HOMA-IR declined by 1.62 (95% CI − 2.79, − 0.46; *p* = 0.007) without such association with changes in PYY hormone. For every twofold increase in postprandial GLP-1 HOMA-IR declined by 0.87 (95% CI − 1.89, 0.15, *p* = 0.098), however this trend did not reach statistical significance.

During 12–36 months, when controlled for the weight change and procedure, HOMA-IR declined by 0.59 (95% CI − 1.10, − 0.10; *p* = 0.023) for every twofold increase in adiponectin level and by 0.91 (95% CI − 1.71, − 0.11; *p* = 0.030) for every twofold increase in postprandial PYY without any comparable association with GLP1 hormone.

During 0–12 months but not during 12–36 months, after controlling for study procedures, weight declined by 13.9 kg (95% CI − 19.17 kg to − 8.66 kg, *p* < 0.0001) for every twofold increase in postprandial PYY and by 6.5 kg (95% CI − 11.42 kg to − 1.54 kg, *p* = 0.011) for every twofold increase in postprandial GLP1 hormone.

#### The associations between changes in RBP4, FGF21 and CRP and changes in insulin resistance

**Figure 2:**
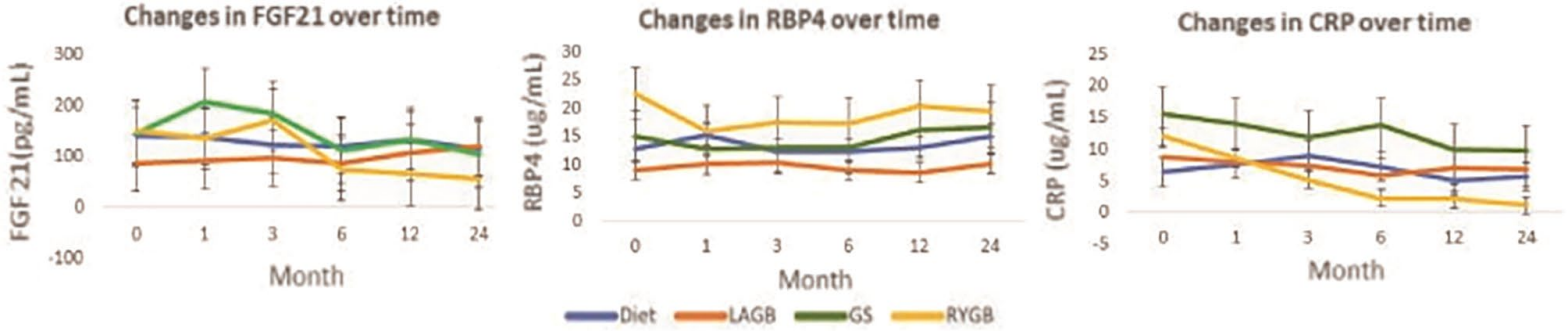
Changes in hormones FGF21, RBP4 and CRP following weight loss procedures.

Successful weight loss post-bariatric surgery during the first 12 months led to significant changes in RBP4, FGF21 and CRP, see Fig. 2. During the second-year post interventions, no further changes in RBP4 or FGF 21 were noted in any of the study groups. The CRP trajectories for RYGB and GS groups remained significantly different in comparison with baseline values and with DIET (*p* < 0.0001 for the two groups). There was no statistically significant association between changes in RBP4, FGF21 and CRP and changes in HOMA-IR during weight loss or weight maintenance phases.

## Discussion

Our study addresses the paucity of literature data comparing the long-term change in insulin resistance after two modalities of weight loss (calorie restriction vs. three different types of bariatric surgery) with bariatric surgeries being more effective than dietary measures in achieving long term glycaemic control. To our knowledge this is the first study which linked post-surgical alterations in postprandial PYY and GLP1 hormones to achieved weight loss as well as to improved insulin resistance beyond first 12 months after weight loss interventions. The present results support the hypothesis that gut hormones and adipose derived factors play a significant role in diverse homeostatic processes and coordinately regulate energy balance and glucose homeostasis.

Congruent with other reports, in comparison with dietary intervention, during the first 12 postoperative months, all surgical groups achieved improvement in their insulin resistance^5^. Furthermore, during this initial phase of rapid weight loss, when weight change was adjusted for, the HOMA-IR values did not differ between surgical groups and DIET. This significant role of weight loss in reductions in glucose, insulin, and HOMA-IR was also reported for 2- and 10-years in the Swedish Obese Subjects (SOS) Study^7^ as well as in the study which compared metabolic benefits of gastric bypass surgery and diet by three-stage hyperinsulinemic euglycemic pancreatic clamp^24^. The achieved improvement in glucose homeostasis was maintained by RYGB and GS groups during their weight stability/regain phase, after the first 12 months post weight loss interventions. Notably the HOMA-IR measurements returned to the physiological values for RYGB and GS groups at the 3-year timepoint.

Although the beneficial effects of bariatric surgery on gluconeogenesis are partly mediated by weight loss, potential neurohormonal mechanisms are not fully understood. The major weight loss at 6 months, in RYGB and GS groups, led to significant alterations in secretion of hormones that are involved in regulating glucose homeostasis. The exaggerated secretions of appetite-regulating gut hormones, adiponectin and inflammatory biomarker CRP were present up to 3 years post RYGB and GS surgeries, however the values of RBP4 and FGF21 hormones were no different to baseline beyond 12 months after these interventions. The LABG and DIET groups did not exhibit statistically significant hormonal alterations.

Interestingly, during the phase of weight stability, for patients without diabetes or those with diabetes not on treatment, their raised post-prandial PYY responses together with their increased adiponectin levels were negatively associated with changes in HOMA-IR, above the effects of surgical procedures and sustained weight loss during this time.

Importantly, PYY hormone with its two endogenous forms: PYY1-36 and PYY3-36.has been well recognized for its role in the reduction in food intake and subsequent weight loss and to a lesser extent for its glycaemic benefits^25,26^. Congruent with our results, a recent murine study using RYGB rat model highlighted a vital role of PYY hormone in recovery of impaired islet secretory function in type 2 diabetes^12^.

Contrary to human RYGB patients, in murine studies, the post bariatric weight loss is achieved mainly through increased energy expenditure rather than decreased food intake^27^. Despite an established role of PYY in metabolism, there is a lack of consensus on its role on insulin secretion in humans. In the previous studies examining human islets, as intravenous 30-min infusion of PYY in healthy individual did not inhibit the acute insulin response to glucose^28^, suggesting that the glycaemic benefit of PYY may be related to its long-term rather than acute effect^28^. Furthermore, a study of subjects examined at 6 months post GS or RYGB surgeries suggested a pivotal effect of PYY hormone in recovery of islet secretory function^15^. In this study, short chain fatty acid propionate, bile acids and IL-22 were molecular mechanisms responsible for triggering of PYY release from human pancreatic islets^15^. Furthermore, evidence from human studies following RYGB surgery showed that chronic exposure of islets to PYY restores normal glucose regulation of insulin and glucagon secretion^12^. Human studies have consistently shown that enhanced postprandial GLP-1 and PYY release are associated with favourable weight loss outcomes after RYGB^29^. Clinical data from randomised controlled trial have shown that the administration of DPP-4 inhibitor, sitagliptin for 12 weeks to diabetic patients improved their both glucose- and non-glucose-stimulated insulin secretion. This occurred together with an increase in PYY (1–36) levels and other incretin hormones, supporting the role of PYY hormone in beneficial effects of DPP-IV inhibition therapy on glycaemic control^30^.

Previous rodent knockout models and human studies reported a key role of PYY hormone in regulation of body weight^31,32^. Therefore, increased PYY hormone would be expected to produce beneficial changes in energy metabolism and reduced appetite^13^. Although our results indicate that enhanced secretion of postprandial PYY facilitate postoperative weight loss, likely through the control of food intake^33^, a comparison of gastric bypass and ileal transposition (IT) in rats showed greater reduction in food intake and weight following RYGB, despite similar PYY enhancement^34^*.* Furthermore, in previous study preoperative responses of GLP-1 and PYY hormones to a mixed meal did not correlate with postoperative weight loss after RYGB surgery^35^.

In several studies, GLP1 hormone, independently from weight loss, has been considered as a significant factor for improved β cell function in type 2 diabetes after bariatric surgery^36^. In particular randomised placebo-controlled trial, performed at 5 months post RYGB surgery, supported the importance of endogenous GLP-1 for postprandial insulin secretion and attenuated glucagon secretion in RYGB operated patients^37^. However, a key role of GLP1 in diabetes remission is not fully supported by animal studies^11^ nor by the results from the present study. It is possible that with a bigger cohort, the association of GLP-1 with HOMA-IR changes might have reached statistical significance. Therefore, in consideration of the current data, the enhanced postprandial GLP1 secretions would contribute to achieved weight loss with the lesser mechanistic effect towards sustained reduction in insulin resistance.

Taken together, our data are congruent with previous observations that PYY and GLP1 hormones, in synergy with other gastrointestinal hormones including ghrelin, may act as potential humoral mediators of weight loss and glycemic improvements after bariatric procedures^31^.

Adiponectin is a metabolically protective hormone with insulin-sensitizing actions in liver and skeletal muscle and anti-inflammatory effects on vascular endothelium^38^. The circulating concentrations of adiponectin correlate negatively with obesity and central adiposity^39,40^. In the present study, all surgical groups but not DIET demonstrated significant increases in adiponectin over the period of 36 months, which paralleled their sustained weight and fat loss. These results are consistent with the literature reporting an increase in adiponectin levels with significant weight loss^41–43^. We have also observed that, for patients without diabetes or those with diabetes not on treatment, the increments in adiponectin levels were negatively associated with changes in HOMA-IR at each time point over 36 months: likely contributing to the long-term improvement in diabetes control. These findings are consistent with epidemiological studies supporting the role of adiponectin as an adipocyte—secreted insulin sensitizing hormone. A Mendelian randomization study provided evidence of a causal effect of increased adiponectin levels in improved insulin sensitivity in humans^44^. Moreover, this relationship between increased adiponectin and insulin sensitivity was shown to be at least partly mediated through a reduction in adipose tissue mass. In the present study, the successful weight loss observed 3 years post GS and RYGB surgeries might have contributed to the long-term improvement in insulin resistance via the modulation of plasma adiponectin levels with subsequently altered pattern in cytokine secretions. The proposed role of adiponectin as an insulin enhancing factor has been supported by a study showing an association between raised adiponectin concentration and improved glucose homeostasis at 5 years post biliopancreatic diversion^45^.

Congruent with some studies, we have observed no statistically significant relationship between changes in RBP4 or FGF21 and insulin resistance after bariatric surgery^46–48^. Decreased CRP levels, related to the loss of body fat, are reflective of subjects’ reduced pro-inflammatory risk^49^.

The strengths of this study are its duration and study design which allowed for prospective examination of postsurgical hormonal changes in fasting and postprandial conditions and their impact on glucogenesis over the two time periods, i.e., in relation to the rapid weight loss (0–12 months) and critically during weight stability over the extended time period (12–36 months). The adjustment for baseline characteristics of study participants, their weight changes and study procedures has allowed us to investigate the associations between examined hormonal indices and HOMA-IR changes above those associated with surgical procedures and the sustained weight loss. A major advantage of this study is the longitudinal assessment of changes in insulin resistance in conjunction with weight loss and detailed analysis of several hormonal indices with evaluation of postprandial responses of PYY and GLP1 hormones. Furthermore, the study analysis accounted for the baseline measurements and hierarchical clustered nature of the longitudinal data with repeated study measurements over the period of 3 years. Given the continuous study outcomes from the random intercept linear mixed effect models analysis, the results from this study are generalizable to subjects undergoing similar weight loss interventions. However, we do acknowledge that these analyses may still not fully explain the mechanistic roles of postsurgical weight and hormone changes on the sustained change in HOMA-IR after bariatric surgery.

The present cohort study has some limitations as due to ethical reasons we did not randomise our subjects with obesity into dietary intervention. Instead, the choice of weight loss procedure was dependent upon patient and physician preference. Thus, our results do not reflect causality between observed hormonal changes and reduction in HOMA-IR as they may be subject to covariate imbalance. We have minimized this bias by adjusting for imbalanced baseline variables.

Although we used HOMA-IR as a surrogate assessment of insulin resistance instead of technically demanding euglycemic hyperinsulinemic clamp (EHC), EHC has been previously shown to correlate well with HOMA-IR values^50^. Considering EHC as a labour intense procedure, to our knowledge, there is a paucity of studies using this technique, which were performed beyond first 6 months post weight loss interventions^51,52^.

Despite the limitation of the relatively small sample size in the RYGB group, the study has produced statistically robust results and therefore the study was sufficiently powered for this group. The RYGB group experienced the greatest change in examined parameters. The study demonstrated significant within group changes in HOMA-IR over the study period and, of particular importance, significant differences in HOMA-IR between groups during the phase of weight stability. Importantly, even with the limited number of RYGB subjects, the study had over 90% power to detect the observed differences between RYGB and diet groups with a type 1 error of 5%.

In conclusion, our results, in line with major studies, demonstrated that bariatric surgery is a more effective long-term weight loss modality than conventional therapy and it results in greater improvement in obesity—related comorbidities such as insulin resistance^6^. We have also shown that RYGB and GS procedures were more effective in comparison with LAGB and Diet procedures in reduction in glycemia and therefore in maintaining longer term improved diabetes control. In addition, we found that RYGB and GS procedures but not LAGB surgeries produced marked responses in postprandial PYY and GLP1, adiponectin and CRP hormones up to 36 months post interventions. These exaggerated hormonal responses were maintained despite stability of weight after the first 12 months post interventions. The current study has suggested a novel mechanism of hormonal regulation of both weight and insulin resistance that has been previously demonstrated to occur in animal models with suggestive but no definitive data in humans. The results from our research link enhanced postprandial responses of PYY and GLP1 hormones to the magnitude of surgically induced weight loss. Furthermore, our results also suggest that long term changes in secretion of gut hormone PYY and adiponectin, in synergy with other hormones that may include GLP1 and CRP, may contribute to improvements in insulin resistance and therefore reduction in future cardiovascular risk following bariatric surgery. Further research into this pathway may lead to novel, nonsurgical treatments for type 2 diabetes. The metabolic roles of RBP4 and FGF21 are likely related to rapid fluctuations in weight or changes in nutritional intake occurring in the first 12 months following weight loss interventions. Further studies involving larger numbers of subjects, as well as ongoing mechanistic studies are needed to fully understand the complex neuroendocrine regulation of weight, appetite and glucose homeostasis in bariatric patients.

## Supplementary Information

Supplementary Table 1.

## Data Availability

All data generated or analysed during this study are included in this published article (and its supplementary information files).

## Abbreviations

CRP: C-reactive protein
FGF 21: Fibroblast growth factor-21
GS: Gastric sleeve surgery
GLP1: Glucagon-like peptide 1
LAGB: Laparoscopic adjustable gastric banding surgery
HOMA-IR: Homeostatic model assessment for insulin resistance
PYY: Polypeptide tyrosine-tyrosine
RBP4: Retinol-binding protein 4
