# Supplementary material for: Effects of bariatric surgery and dietary intervention on insulin resistance and appetite hormones over a 3 year period
Source: Sci Rep. 2023 Apr 13;13:6032. doi: 10.1038/s41598-023-33317-6 (PMC10102182; doi:10.1038/s41598-023-33317-6)
Supplement: Supplementary file 1 — Supplementary Table 1. [file 41598_2023_33317_MOESM1_ESM.docx]

| Supplementary Table 1: Descriptive results at each time point. | | | | | | | |
| --- | --- | --- | --- | --- | --- | --- | --- |
| **Variables** | **Baseline** | **1 month** | **3 months** | **6 months** | **12 months** | **24 months** | **36 months** |
| **Diet** |  |  |  |  |  |  |  |
| Weight (kg) | 110.9 (6.51), ^16^ | 108.6 (6.10), ^16^ | 106.1 (5.00), ^15^ | 1105.5 (4.86),^15^ | 106.4 (6.20), ^14^ | 111.5 (37.76), ^13^ |  |
| Fat mass (kg) | 49.09 (2.39), ^15^ |  |  | 42.74 (4.15), ^6^ | 45.54 (2.65), ^13^ | 47.39 (3.45), ^10^ |  |
| Lean mass (kg) | 54.32 (2.41), ^15^ |  |  | 56.58 (4.59), ^6^ | 53.82 (2.67), ^13^ | 56.21 (3.20), ^10^ |  |
| BMI (kg/m^2^) | 38.13 (1.64), ^16^ | 37.31 (1.36), ^16^ | 36.67 (1.19), ^15^ | 36.20 (1.21), ^15^ | 36.14 (1.39), ^14^ | 37.85 (7.48), ^13^ |  |
| Waist (cm) | 122.1 (3.56), ^16^ | 117.4 (3.53), ^16^ | 116.1 (3.39). ^15^ | 115.7 (3.16), ^15^ | 113.0 (3.39), ^13^ | 117.6 (19.96), ^13^ |  |
| Fasting glucose (mmol/L) | 6.95 (0.88), ^15^ | 6.36 (0.55), ^16^ | 6.84 (0.87), ^15^ | 6.85 (1.01), ^15^ | 6.11 (0.78), ^14^ | 5.58 (0.69), ^13^ |  |
| HbA1C (%) | 6.45 (0.38), ^15^ | 6.35 (0.33), ^16^ | 6.50 (0.37), ^15^ | 6.56 (0.48), ^15^ | 6.04 (0.34), ^14^ | 5.85 (0.66), ^13^ |  |
| Adiponectin (ng/mL) | 6780 (1285), ^14^ | 7276 (1406), ^15^ | 7034 (1337), ^13^ | 7621 (1623), ^15^ | 9669 (2974), ^13^ | 6130 (4653), ^12^ |  |
| FGF21 (pg/mL) | 138.4 (29.37), ^16^ | 137.1 (22.79), ^15^ | 120.6 (17.08), ^13^ | 118.8 (23.49), ^14^ | 130.5 (33.17), ^14^ | 113.6 (24.96), ^11^ |  |
| RBP4 (ug/mL) | 12.70 (0.96), ^16^ | 15.24 (1.73), ^15^ | 12.28 (1.18), ^13^ | 12.36 (1.08), ^14^ | 12.84 (1.01), ^14^ | 14.97 (0.78), ^11^ |  |
| CRP (ug/mL) | 6.45 (1.33), ^16^ | 7.61 (1.54), ^15^ | 8.99 (2.35), ^13^ | 7.11 (1.49), ^14^ | 5.08 (1.04), ^14^ | 5.66 (1.14), ^11^ |  |
| PYY- Fasting* (pg/ml) | 105 (72-129),^16^ |  | 103 (79-133),^16^ | 111 (83-142)^15^ | 83 (66-132),^14^ | 83 (44-127),^13^ |  |
| Post-prandial PYY*  (pg/ml), | 135 (108-175),^16^ |  |  | 144 (113-181),^7^ | 118 (73-142),^14^ |  |  |
| Fasting GLP1* (pM/ml) | 8.1 (7.6-12),^16^ |  | 8.3 (7.5-8.9)^16^ | 8.2 (7.5-8.9),^15^ | 8.2 (7.4-11.1),^14^ | 6.7 (4.7-13.5),^13^ |  |
| Post-prandial GLP1* (pM/ml) | 8.8 (7.8-14).^16^ |  |  | 9.3 (8.7-13.4),^7^ | 10.3 (8.2-20.9),^14^ |  |  |
| Insulin (mU/L) | 16.35 (2.26), ^10^ |  |  | 9.95 (1.49),^11^ | 15.29 (2.60), ^11^ | 17.81 (3.25), ^9^ |  |
| HOMA-IR | 5.81 (1.55), ^10^ |  |  | 3.67 (1.22),^11^ | 4.69 (1.22), ^11^ | 4.59 (1.05), ^9^ |  |
| **Gastric Band** |  |  |  |  |  |  |  |
| Weight (kg) | 105.0 (4.97), ^11^ | 98.09 (4.41), ^11^ | 94.00 (4.33), ^11^ | 91.73 (3.45), ^11^ | 88.90 (3.07), ^10^ | 90.0 (11.8), ^9^ | 85.4 (12.1), ^8^ |
| Fat mass (kg) | 51.28 (2.79), ^11^ |  |  | 40.44 (2.77), ^9^ | 39.28 (2.00), ^10^ | 40.61 (2.57), ^9^ | 39.1 (8.2), ^8^ |
| Lean mass (kg) | 50.50 (2.14), ^11^ |  |  | 49.00 (2.16), ^9^ | 47.69 (2.15), ^10^ | 47.19 (2.47), ^9^ | 45.3 (7.6), ^8^ |
| BMI (kg/m^2^) | 37.73 (1.36), ^11^ | 35.00 (1.32), ^11^ | 33.91 (1.23), ^11^ | 33.00 (1.07), ^11^ | 32.40 (0.92), ^10^ | 33.67 (3.64), ^9^ | 31.61 (3.4), ^8^ |
| Waist (cm). | 110.9 (2.19), ^11^ | 107.4 (2.18), ^11^ | 100.9 (2.34), ^11^ | 102.0 (2.71), ^11^ | 99.50 (2.32), ^10^ | 99.22 (8.41), ^9^ |  |
| Fasting glucose (mmol/L) | 5.08 (0.23), ^11^ | 5.00 (0.34), ^11^ | 5.03 (0.18), ^11^ | 4.83 (0.19), ^11^ | 4.91 (0.18), ^10^ | 5.13 (1.47^), 9^ |  |
| HbA1C (%) | 5.56 (0.16), ^11^ | 5.48 (0.16), ^11^ | 5.36 (0.08), ^11^ | 5.45 (0.14), ^11^ | 5.36 (0.15), ^10^ | 5.60 (1.11), ^9^ |  |
| Adiponectin (ng/mL) | 6863 (707.4), ^11^ | 8746 (804.0), ^10^ | 7775 (873.6), ^11^ | 8205 (850.4), ^11^ | 9034 (1184), ^9^ | 10224 (7688), ^9^ |  |
| FGF21 (pg/mL) | 85.06 (19.01), ^11^ | 91.38 (27.43), ^10^ | 95.28 (19.15),^11^ | 84.28 (18.80), ^11^ | 105.7 (24.33), ^10^ | 117.5 (39.82), ^6^ |  |
| RBP4 (ug/mL) | 8.92 (0.77), ^11^ | 9.96 (0.94), ^10^ | 10.32 (1.20), ^11^ | 8.94 (0.83), ^11^ | 8.48 (0.82), ^10^ | 10.08 (0.81), ^6^ |  |
| CRP (ug/mL) | 8.80 (3.02), ^11^ | 7.91 (3.19), ^10^ | 7.37 (2.87),^11^ | 5.81 (1.84), ^11^ | 6.98 (2.66), ^10^ | 6.77 (2.51), ^6^ |  |
| Fasting PYY* (pg/ml) | 65 (47-111), ^11^ |  | 103 (79-133),^11^ | 98.5 (63-141),^11^ | 67 (61-121),^10^ | 61 (49-100),^9^ |  |
| Post-prandial PYY*(pg/ml) | 94 (64-159),^11^ |  |  | 111 (58-156, ^6^ | 148 (69-164),^6^ |  |  |
| Fasting GLP1* (pM/ml) | 6.4 (6.1-10.0),^11^ |  | 8.3 (7.5-8.9),^11^ | 7.2 (5.9-9.5),^11^ | 7.0 (5.0-8.1),^10^ | 3.5 (2.4-3.9),^9^ |  |
| Post-prandial GLP1* (pM/ml) | 7.6 (6.4-9.6),^11^ |  |  | 7.6 (6.4-9.6),^6^ | 7.0 (5.2-9.5),^6^ |  |  |
| Insulin (mU/L) | 15.88 (3.53), ^9^ |  |  | 9.48 (1.57), ^9^ | 10.06 (1.88), ^9^ | 10.70 (2.65), ^5^ |  |
| HOMA-IR | 3.60 (0.85), ^9^ |  |  | 2.05 (0.38), ^9^ | 2.18 (0.43), ^9^ | 2.48 (0.52), ^5^ |  |
| **Gastric Sleeve** |  |  |  |  |  |  |  |
| Weight (kg) | 125.3 (3.68), ^21^ | 110.6 (3.40), ^20^ | 103.0 (3.31), ^20^ | 95.65 (3.03), ^20^ | 92.65 (3.15), ^20^ | 92.7 (12.7), ^19^ | 93.6 (13.5), ^18^ |
| Fat mass (kg) | 56.77 (1.89), ^19^ |  |  | 38.07 (2.33), ^16^ | 36.32 (2.54), ^20^ | 36.9 (9.1), ^19^ | 38.2 (7.7), ^18^ |
| Lean mass (kg) | 60.45 (1.82), ^19^ |  |  | 55.61 (1.88), ^16^ | 54.33 (1.78), ^20^ | 53.2 (8.1), ^19^ | 53.1 (8.6), ^18^ |
| BMI (kg/m^2^) | 42.52 (1.15), ^21^ | 37.80 (1.08), ^20^ | 34.75 (0.97), ^20^ | 32.45 (0.90), ^20^ | 31.40 (0.90), ^20^ | 31.72 (3.73), ^18^ | 30.46 (7.4), ^17^ |
| Waist (cm) | 132.3 (2.74), ^21^ | 118.2 (2.22), ^20^ | 112.6 (2.48), ^20^ | 107.1 (2.28), ^20^ | 102.9 (2.16), ^20^ | 103.8 (12.15), ^18^ | 105.6 (14.78), ^16^ |
| Fasting glucose (mmol/L) | 5.61 (0.30), ^21^ | 5.24 (0.21), ^20^ | 5.00 (0.23), ^20^ | 4.74 (0.19), ^20^ | 4.75 (0.14), ^20^ | 5.23 (2.03), ^19^ | 5.33 (2.09), ^17^ |
| HbA1C (%) | 6.07 (0.15), ^21^ | 5.72 (0.09), ^20^ | 5.58 (0.08), ^20^ | 5.50 (0.09), ^20^ | 5.32 (0.08), ^20^ | 5.43 (0.81), ^19^ | 5.77 (1.27), ^15^ |
| Adiponectin  (ng/mL) | 7083 (779.6), ^19^ | 7929 (974.5), ^19^ | 8925 (813.5), ^19^ | 10272 (921.3), ^18^ | 10942 (1415), ^19^ | 11116 (11574), ^19^ | 14686 (8800), ^17^ |
| FGF21 (pg/mL) | 143.4 (29.56), ^21^ | 206.1 (35.29), ^20^ | 181.8 (46.09), ^19^ | 111.0 (25.59), ^20^ | 130.4 (31.87), ^20^ | 109.4 (32.00), ^15^ |  |
| RBP4 (ug/mL) | 14.89 (2.71), ^21^ | 12.65 (2.09), ^20^ | 13.04 (2.18), ^19^ | 12.98 (2.04), ^20^ | 15.99 (3.10), ^20^ | 17.13 (3.91), ^15^ |  |
| CRP (ug/mL) | 15.55 (2.73), ^21^ | 13.91 (3.59), ^20^ | 11.89 (2.97), ^19^ | 13.86 (3.19), ^20^ | 9.83 (2.58), ^20^ | 10.17 (3.43), ^15^ |  |
| Fasting PYY* (pg/ml) | 105 (82-152), ^21^ |  | 95.5 (74-141), ^20^ | 101 (69-143),^21^ | 97 (56-135),^20^ | 76 (47-123),^15^ | 105 (88-129),^17^ |
| Post-prandial PYY* (pg/ml) | 134 (105-178), ^21^ |  |  | 248 (146-315)^15^ | 205 (131-259),^20^ | 259 (176-346)^,4^ | 204 (158-313),^17^ |
| Fasting GLP1* (pM/ml) | 7.5 (6.5-8.0), ^21^ |  | 7.4 (6.8-7.9), ^20^ | 7.1 (6.6-7.6),^21^ | 6.7 (5.9-7.9),^20^ | 6.7 (4.4-10.6),^15^ | 3.9 (3.2-4.2),^17^ |
| Post-prandial GLP1* (pM/ml) | 8.1 (7.5-8.7), ^21^ |  |  | 8.2 (7.0-9.9)^15^ | 8.5 (6.4-10.9),^20^ | 5.7 (2.8-11.4),^4^ | 5.1 (4.4-7.5),^17^ |
| Insulin (mU/L) | 24.44 (4.21), ^17^ |  |  | 9.07 (1.45), ^18^ | 9.25 (1.22), ^17^ | 9.76 (1.19), ^15^ | 6.05 (4.7), ^12^ |
| HOMA | 6.79 (1.82), ^17^ |  |  | 2.09 (0.48), ^18^ | 2.00 (0.30), ^17^ | 2.12 (0.33), ^15^ | 1.25 (1.15), ^11^ |
| **Gastric Bypass** |  |  |  |  |  |  |  |
| Weight (kg) | 113.0 (15.9), ^7^ | 97.57 (6.07), ^7^ | 87.6 (14.4), ^7^ | 79.2 (12.7), ^7^ | 73.7 (12.4), ^7^ | 74.6 (13.1), ^7^ | 75.0 (13.7), ^7^ |
| Fat mass (kg) | 54.9 (13.4), ^7^ |  |  | 29.1 (11.2), ^7^ | 24.2 (7.8), ^7^ | 26.0 (8.4), ^6^ | 29.1 (7.4), ^7^ |
| Lean mass (kg) | 54.3 (9.7), ^7^ |  |  | 48.6 (9.80), ^7^ | 48.3 (10.2), ^7^ | 44.8 (9.2), ^6^ | 46.1 (10.0), ^7^ |
| BMI (kg/m^2^) | 42.29 (2.90), ^7^ | 36.43 (2.63), ^7^ | 32.86 (2.51), ^7^ | 29.43 (2.11), ^7^ | 27.57 (1.69), ^7^ | 27.86 (4.81), ^7^ | 28.57 (4.24), ^7^ |
| Waist (cm) | 119.7 (4.65), ^7^ | 107.9 (3.86), ^7^ | 100.0 (4.15), ^7^ | 94.00 (2.60), ^7^ | 89.43 (4.10), ^7^ | 88.93 (12.65), ^7^ | 92.64 (9.94), ^7^ |
| Fasting glucose (mmol/L) | 7.61 (1.57), ^7^ | 5.36 (0.29), ^7^ | 4.90 (0.25), ^7^ | 4.74 (0.21), ^7^ | 5.01 (0.36), ^7^ | 5.09 (1.1), ^7^ | 5.27 (1.16), ^7^ |
| HbA1C (%) | 6.76 (0.82), ^7^ | 5.66 (0.42), ^7^ | 5.29 (0.29), ^7^ | 5.14 (0.20), ^7^ | 5.07 (0.28), ^7^ | 5.19 (0.81), ^7^ | 5.33 (0.91), ^7^ |
| Adiponectin  (ng/mL) | 7835 (1880), ^7^ | 9226 (1431), ^7^ | 9908 (1140), ^7^ | 10762 (1885), ^6^ | 15877 (3360), ^5^ | 18702 (23332), ^7^ | 15500 (21400), ^7^ |
| FGF21 (pg/mL) | 148.6 (63.06), ^7^ | 134.3 (23.64), ^6^ | 169.7 (48.04), ^7^ | 73.63 (15.90), ^7^ | 64.33 (15.86), ^7^ | 66.46 (11.60), ^3^ |  |
| RBP4 (ug/mL) | 22.51 (3.52), ^7^ | 15.91 (2.13), ^6^ | 17.40 (2.47), ^7^ | 17.17 (2.34), ^7^ | 20.21 (1.87), ^7^ | 17.46 (1.13), ^3^ |  |
| CRP (ug/mL) | 11.99 (2.39), ^7^ | 8.52 (2.12), ^6^ | 5.09 (1.39), ^7^ | 2.19 (0.49), ^7^ | 2.05 (0.57), ^7^ | 1.01 (0.30), ^3^ |  |
| Fasting PYY* (pg/ml) | 84 (67-119),^7^ |  | 118.5 (90-173),^7^ | 113 (97-130),^7^ | 118 (89-152),^7^ | 177 (136-185),^7^ | 113 (106-245),^7^ |
| Post-prandial PYY* (pg/ml) | 121 (77-133),^7^ |  |  | 305 (232-311),^5^ | 245 (206-318),^7^ | 308 (304-329)^5^ | 304 (261-356),^7^ |
| Fasting GLP1* (pM/ml) | 5.7 (5.0-6.1),^6^ |  | 6.1 (5.8-6.6),^7^ | 6.1 (6.0-6.9),^5^ | 4.8 (3.7-5.7),^7^ | 3.1 (2.1-4.4),^7^ | 2.3 (1.5-6.6),^7^ |
| Post-prandial GLP1* (pM/ml) | 6.5 (5.3-7.0),^7^ |  |  | 17.5 (10.1-19.0),^5^ | 8.3 (5.2-14.8),^7^ | 8.6 (4.5-12.4)^,5^ | 11.3 (10.6-12.1),^7^ |
| Insulin (mU/L) | 21.80 (12.41), ^4^ |  |  | 7.29 (3.18), ^3^ | 4.35 (0.76), ^4^ | 4.20 (1.20), ^3^ | 3.7 (1.25), ^3^ |
| HOMA | 6.36 (4.15), ^4^ |  |  | 1.50 (0.69), ^3^ | 0.87 (0.13), ^4^ | 0.90 (0.27), ^3^ | 0.8 (0.25), ^3^ |

*Results presented as mean(SEM) unless otherwise indicated.,* **median (IQR), number of participants.*
